# Identification and analysis of microRNA editing events in recurrent bladder cancer based on RNA sequencing: MicroRNA editing level is a potential novel biomarker

**DOI:** 10.3389/fgene.2022.984279

**Published:** 2022-09-19

**Authors:** Jia-Xin Qin, Xing Liu, Xin-Lei Wang, Guang-Yue Wang, Qing Liang, Yang Dong, Kun Pang, Lin Hao, Liang Xue, Yan Zhao, Zheng-Xiang Hu, Rui Li, Qian Lv, Liu Chao, Fan-Lai Meng, Zhen-Duo Shi, Cong-Hui Han

**Affiliations:** ^1^ Department of Urology, Xuzhou Clinical College of Xuzhou Medical University, Xuzhou, China; ^2^ Department of Urology, Xuzhou Central Hospital, Xuzhou, China; ^3^ Graduate School of Bengbu Medical College, Bengbu, China; ^4^ Graduate School of Jinzhou Medical College, Jinzhou, China; ^5^ Central Laboratory, Xuzhou Central Hospital, Xuzhou, China; ^6^ Department of Urology, The Suqian Affiliated Hospital of Xuzhou Medical University School, Suqian, China; ^7^ Department of Pathology, The Suqian Affiliated Hospital of Xuzhou Medical University School, Suqian, China; ^8^ School of Life Sciences, Jiangsu Normal University, Xuzhou, China; ^9^ Department of Urology, Heilongjiang Provincial Hospital, Harbin, China

**Keywords:** RNA sequencing, bladder cancer, tumor recurrence, microRNA editing, consensus molecular subtype classification

## Abstract

**Background:** With the continued advancement of RNA-seq (RNA-sequencing), microRNA (miRNA) editing events have been demonstrated to play an important role in different malignancies. However, there is yet no description of the miRNA editing events in recurrent bladder cancer.

**Objective:** To identify and compare miRNA editing events in primary and recurrent bladder cancer, as well as to investigate the potential molecular mechanism and its impact on patient prognosis.

**Methods:** We examined the mRNA and miRNA transcriptomes of 12 recurrent bladder cancer cases and 13 primary bladder cancer cases. The differentially expressed mRNA sequences were analyzed. Furthermore, we identified the differentially expressed genes (DEGs) in recurrent bladder cancer. The Gene Ontology (GO) functional enrichment analyses on DEGs and gene set enrichment analysis were performed. The consensus molecular subtype (CMS) classification of bladder cancer was identified using the Consensus MIBC package in R (4.1.0); miRNA sequences were then further subjected to differentially expressed analysis and pathway enrichment analysis. MiRNA editing events were identified using miRge3.0. miRDB and TargetScanHuman were used to predict the downstream targets of specific differentially edited or expressed miRNAs. The expression levels of miR-154-5p and ADAR were validated by RT-qPCR. Finally, survival and co-expression studies were performed on the TCGA-BLCA cohort.

**Results:** First, the mRNA expression levels in recurrent bladder cancer changed significantly, supporting progression via related molecular signal pathways. Second, significantly altered miRNAs in recurrent bladder cancer were identified, with miR-154-5p showing the highest level of editing in recurrent bladder cancer and may up-regulate the expression levels of downstream targets HS3ST3A1, AQP9, MYLK, and RAB23. The survival analysis results of TCGA data revealed that highly expressed HS3ST3A1 and RAB23 exhibited poor prognosis. In addition, miR-154 editing events were found to be significant to CMS classification.

**Conclusion:** MiRNA editing in recurrent bladder cancer was detected and linked with poor patient prognosis, providing a reference for further uncovering the intricate molecular mechanism in recurrent bladder cancer. Therefore, inhibiting A-to-I editing of miRNA may be a viable target for bladder cancer treatment, allowing current treatment choices to be expanded and individualized.

## 1 Introduction

Bladder cancer (BC) is one of the most common urological carcinomas, with significant morbidity in patients worldwide (Richters et al., 2020). Most BCs are urothelial carcinomas (Bladder Urothelial Carcinoma, BLCA), which are classified into two clinical and prognostic subtypes: non-muscle-invasive bladder cancers (NMIBCs) and muscle-invasive bladder cancers (MIBCs). NMIBC accounts for about 75% of cases of BC (Babjuk et al., 2019). The biological mechanisms of bladder cancer are exceedingly complicated, with various traits including heterochrony and heterotopy, high recurrence, multiple tumors, progression to invasive bladder cancer, and metastasis (van Rhijn et al., 2009). As a result, unraveling novel molecular mechanisms of BC progression and discovering novel tumor markers capable of predicting survival and recurrence are urgently required.

RNA editing alters the nucleotide sequence of an RNA molecule post transcription, which can occur in coding or non-coding regions, increasing transcriptome variability and proteome diversity (Eisenberg, 2021). Adenosine to inosine (A-to-I) and cytidine to uridine (C-to-U) editing are two types of RNA editing, with A-to-I being the most common (Leong et al., 2019). A-to-I RNA editing is catalyzed by members of the ADAR family of dsRNA binding proteins (adenosine deaminases acting on RNA), which comprises three constitutive isoforms in humans, labelled ADAR (ADAR1), ADARB1 (ADAR2), and ADARB2 (ADAR3). With the development of RNA deep sequencing technologies (RNA-seq), recent evidence indicates that most editing occurs on non-coding RNAs (Li et al., 2009).

MicroRNAs (miRNAs) are endogenous non-coding RNA molecules with 22 nucleotide sequences that play regulatory roles in developmental, physiological, and pathological processes, including cancer (Saliminejad et al., 2019). The first step in miRNA synthesis is RNA polymerase II, which catalyzes the template strand to create pri-miRNA with a cap structure (7MGpppG) and a polyadenylated tail (Lee et al., 2004). The pre-miRNA is then processed by a multiprotein complex that includes cofactor Pasha from RNA polymerase III nuclease DROSHA (Denli et al., 2004), which is exported to the cytoplasm via the nuclear pore complex and subsequently cleaved into double-stranded miRNA duplexes by Dicer of another RNA polymerase III nuclease. Double-stranded miRNAs generate two types of mature miRNAs, one from the 5P chain and the other from the 3P chain, which combines with Argonaute (AGO) family proteins into an RNA-induced silencing complex (RISC) and directs their binding to mRNA (Kobayashi and Tomari, 2016). Editing in pri-miRNA, pre-miRNA, and mature miRNA can influence miRNA synthesis and target identification, with far-reaching consequences (Shevchenko and Morris, 2018). Let-7 pri-miRNA editing, for example, impairs let-7 family miRNA biogenesis and increases progenitor self-renewal capacity in blast crisis chronic myeloid leukaemia (BC CML), resulting in malignant reprogramming of progenitors into blast crisis leukaemia stem cells (BC LSCs) (Zipeto et al., 2016). Research evidence shows that miR-589-3p editing is nearly complete in normal brain tissue, but continues to decline in glioblastoma due to a drop in ADAR 2 enzyme levels; this explains the increase of unedited miR-589-3p. The switch from PCDH 9 (tumor suppressor) to ADAM 12, which can encode metalloproteinase 12, promotes glioblastoma invasion and development (Cesarini et al., 2018).

Researchers have gained a deeper understanding of miRNA editing in recent years, with increasing evidence that miRNA editing is prevalent in different types of human cancers, including liver hepatocellular carcinoma (LIHC), lung adenocarcinoma (LUAD), kidney renal clear-cell carcinoma (KIRC), blast crisis chronic myeloid leukaemia (BC CML), glioblastoma and thyroid carcinoma (THCA), among others. (Liao et al., 2020). Subsequent studies revealed that miRNA editing is linked to cancer onset, progression, and prognosis (Gu et al., 2019; Romano et al., 2020), suggesting that it could be utilized as a prognostic marker and therapeutic target (Voss et al., 2021). However, miRNA editing is rarely documented in BC. Yumeng Wang et al. (2017) discovered that miRNA editing changed significantly in BC (Wang et al., 2017), but they did not investigate its link with recurrence, progression, and prognosis. Because of the high recurrence rate of BC, we believe it is critical to investigate the miRNA editing events that occur between recurrent and primary BC.

In this study, we comprehensively analyzed and compared the miRNA editing level of 12 recurrent BC and 13 primary BC cases by RNA sequencing, including mRNA expression analysis, miRNA expression analysis, and miRNA editing analysis. Our findings indicate that the degree of miRNA editing in recurrent BC is significantly higher and is associated with poor patient survival. This finding could aid in the investigation of pathogenesis and mechanisms, and provide potential biomarkers and novel therapeutic targets for BC.

## 2 Methods

### 2.1 Patient sample collection

The study was performed at the Urology Surgery Department of Xuzhou Central Hospital, Xuzhou 221009, PR China. The subjects were recruited from 2021.06 to 2021.08. Tissue samples were acquired from bladder cancer patients who underwent laparoscopic radical cystectomy, partial cystectomy, or transurethral resection of bladder tumor. The tissues were immediately cleaned with sterile normal saline following surgery and stored at −80°C until detection. A total of 25 patients with bladder cancer were included, and all protocols were approved by the Ethical Committee of the Xuzhou Central Hospital (EC. XZXY-LI-20200708-024). All subjects signed a written consent form.

### 2.2 mRNA sequencing experiments and analysis

#### 2.2.1 mRNA sequencing

Total RNA was extracted using the Quick-RNA MicroPrep Kit (Zymo Research, CA, United States). The concentration and integrity of mRNA were measured by Nanodrop2000 (Thermofisher, Waltham, MA). The cDNA library was prepared with the NEBNext Ultra RNA with Poly-A selection kit and sequenced on an Illumina Hi-Seq 4000.

#### 2.2.2 mRNA-seq analysis

Kallisto (https://pachterlab.github.io/kallisto/) was used for pseudo alignment of the mRNA raw counts. DESeq2 bioconductor package was used to evaluate differential gene expression with |fold change| >2 and *p* < 0.05 genes with >1 count per million (Love et al., 2014) (R 4.1.0). After Benjamini–Hochberg correction for multi-testing, any gene with a *p*-value less than False Discovery Rate (FDR) was judged significantly differentially expressed under the test conditions as compared to the controls. Further pathway enrichment analysis was performed with EnrichR and clusterprofiler (Yu et al., 2012). The gene set enrichment analysis (GSEA) was performed with javaGSEA2-3.0 (Subramanian et al., 2005).

#### 2.2.3 Bladder cancer CMS classification

The expression matrices of each bladder cancer sample were extracted, organized, and then used for CMS classification analysis. The consensus MIBC (Kamoun et al., 2020) package based on R (4.1.0) was used to identify the CMS classification of each bladder cancer sample and then used for the downstream analysis.

### 2.3 MicroRNA sequencing experiments and analysis

#### 2.3.1 Small RNA sequencing and data analysis

A small RNA-seq library was constructed with NEBNext Multiplex Small RNA Library Prep Kit for Illumina (#7560S). Small RNA-seq data were analyzed with the sRNAnalyzer pipeline (Wu et al., 2017). Adapter sequences were input to the pipeline to remove adapter contamination. The cleaned reads were then mapped to the human miRNA database to generate a count matrix, and thereafter DEseq2 was used to identify differentially expressed miRNA. *p*-value less than 0.05 denoted significantly expressed miRNA. The miRNA targets were predicted using the R package ‘Multimir’ (1.10.0). Finally, EnrichR was used to perform the pathway enrichment analysis.

### 2.3.2 MiRNA A-to-I editing and downstream analysis

miRge3.0 (Patil and Halushka, 2021) was used to evaluate the miRNA editing events in bladder cancer samples after the adapters were trimmed. Using the default parameters, the A-to-I editing ratio and locations were determined. Percentage A-to-I editing (%) was calculated by dividing the number of edited miRNAs by the total number of miRNAs expressed with a read count greater than equal to 10. An A-to-I ratio >2% was employed as the filtering cutoff to remove any potential editing noise. MiRNA target prediction tools, including miRDB (http://mirdb.org/) and TargetScanHuman (https://www.targetscan.org/vert_80/), were used to predict the downstream targets of specific differentially edited or expressed miRNAs.

### 2.4 Real-time quantitative PCR (RT‐qPCR)

Real-time quantitative PCR (RT-qPCR) was performed to validate the expression of specific mature miRNAs using pre-designed stem-loop primers (Suzhou GENEWIZ Co., Ltd.). cDNA was synthesized from 10 ng of total RNA using miScript II RT kit (Qiagen) according to the manufacturer’s instructions. For mRNA, 1 μg of total RNA was used as a template to synthesize cDNA using the First Strand cDNA Synthesis Kit (Invitrogen K1622) according to the manufacturer’s protocol. The relative amount of each substrate was calculated by the 2−ΔΔCT method (Livak and Schmittgen, 2001). The small endogenous nuclear RNA U6 and GAPDH were used as controls for the normalization of mature miRNAs and mRNAs. The sequences of the primers used supplied by GENEWIZ were as follows: U6 Forward, 5′- CTCGCTTCGGCAGCACA -3′ and reverse, 5′- AAC​GCT​TCA​CGA​ATT​TGC​GT -3′; has-miR-154-5p F-primer, 5′- GGT​GTG​TAG​GTT​ATC​CGT​GTT​G -3′; human GAPDH Forward, 5′- GCG​AGA​TCC​CTC​CAA​AAT​CAA -3′ and reverse, 5′- GTT​CAC​ACC​CAT​GAC​GAA​CAT -3′; human ADAR F-primer, 5′- GCG​ACC​AGA​CCA​TTG​ATT​CC -3′ and reverse, 5′- TGG​TAC​CTG​AGC​TGT​CTG​TG -3′.

### 2.5 The cancer genome atlas survival analysis of specific genes

The TCGA bladder cancer cohort was used for the survival analysis in this study. Moreover, UALCAN, CbioPortal, and GEPIA (Chandrashekar et al., 2017; Tang et al., 2017) (1.0 and 2.0) were used for pan-cancer analysis and expression analysis of targets of differentially expressed/edited miRNAs. Survival and co-expression analyses were performed using the GEPIA web interface.

### 2.6 Statistical analysis

The False Discovery Rate (FDR) correction was used to get the p-adj value in all expression matrix data (RNA-seq, small-RNA-seq). Data (mean ± SE) were assessed with a Student’s t-test, and a *p*-value < 0.05 was considered statistically significant.

## 3 Results

### 3.1 Patient information

Table 1 shows the clinicopathological characteristics of enrolled patients. All patients in this study had urothelial carcinoma. A total of 25 patients, 1 female and 24 males, with ages ranging from 58 to 92 were enrolled, with 12 samples having NMIBC and 13 samples having MIBC. Patients were divided into two groups based on their clinical diagnosis: the primary bladder cancer group (*n* = 13) and the recurrent bladder cancer group (*n* = 12). All patients had laparoscopic radical cystectomy (LRC, *n* = 6) or partial cystectomy (PC, *n* = 3), as well as transurethral resection of bladder tumor (TURBT, *n* = 16), including 11 cases of low pathological grade (LG) and 14 cases of high pathological grade (HG).

**TABLE 1:** Patients information.

Patients information							
Category	Number	Gender	Age	Pathologic tumor staging	Grade	Surge	RNA-seq
RE	RE1	M	70	MIBC	HG	LRC	miRNA + mRNA
RE	RE2	M	92	MIBC	HG	LRC	miRNA + mRNA
RE	RE3	M	87	MIBC	HG	PC	miRNA + mRNA
RE	RE4	M	74	MIBC	HG	TURBT	miRNA
RE	RE5	M	77	MIBC	HG	PC	miRNA
RE	RE6	M	66	MIBC	HG	TURBT	miRNA
RE	RE7	M	67	NMIBC	LG	TURBT	miRNA
RE	RE8	M	86	NMIBC	LG	TURBT	miRNA
RE	RE9	M	64	MIBC	HG	TURBT	miRNA
RE	RE11	F	68	MIBC	HG	LRC	miRNA + mRNA
RE	RE13	M	71	NMIBC	LG	LRC	miRNA
RE	RE15	M	58	MIBC	HG	TURBT	miRNA + mRNA
NEW	NEW1	M	78	MIBC	HG	TURBT	miRNA
NEW	NEW2	M	71	MIBC	HG	TURBT	miRNA
NEW	NEW3	M	64	MIBC	HG	TURBT	miRNA
NEW	NEW4	M	72	NMIBC	HG	TURBT	miRNA
NEW	NEW5	M	58	MIBC	HG	TURBT	miRNA
NEW	NEW6	M	69	NMIBC	LG	TURBT	miRNA + mRNA
NEW	NEW7	M	57	NMIBC	LG	TURBT	miRNA + mRNA
NEW	NEW8	M	71	NMIBC	LG	PC	miRNA + mRNA
NEW	NEW9	M	81	NMIBC	LG	TURBT	miRNA + mRNA
NEW	NEW11	M	59	NMIBC	LG	LRC	miRNA + mRNA
NEW	NEW12	M	67	NMIBC	LG	TURBT	miRNA
NEW	NEW13	M	68	NMIBC	LG	TURBT	miRNA
NEW	NEW14	M	58	NMIBC	LG	LRC	miRNA

NEW, primary bladder cancer; RE, recurrent bladder cancer; F, female; M, male; NMIBC, non-muscle invasive bladder cancer; MIBC, muscle-invasive bladder cancer; LG, low pathological grade; HG, high pathological grade; TURBT, transurethral resection of the bladder tumor; LRC, laparoscopic radical cystectomy; PC, partial cystectomy.

### 3.2 Identification of differentially expressed genes

**FIGURE 1:**
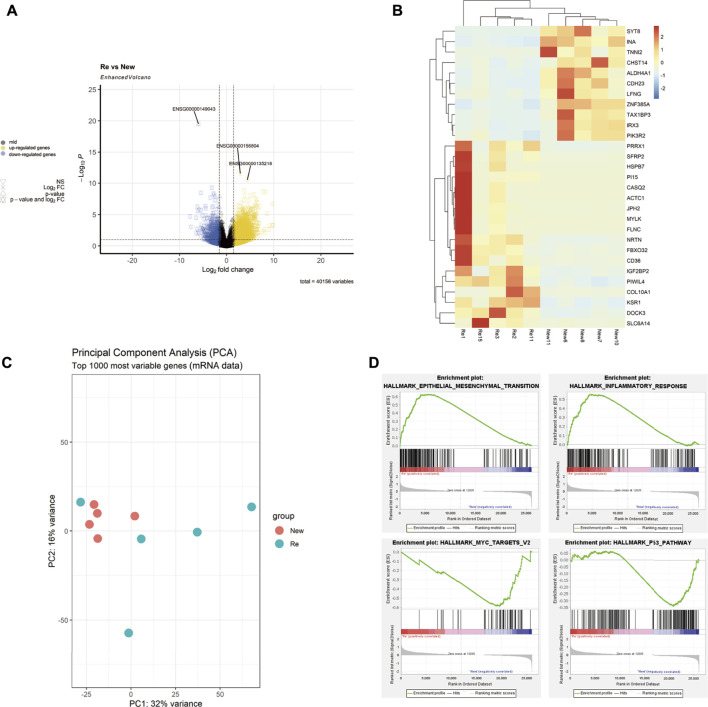
Description of mRNA-seq analysis results of 5 recurrent vs. 5 primary tumors. **(A)** A Volcano plot showing the differentially expressed genes (DEG) in recurrent versus primary bladder tumors (Re: Recurrent, New: Primary), DEG: | Log2Foldchange | > 1, P-FDR < 0.05. **(B)** A Heatmap showing the top 30 significantly differentially expressed genes in the recurrent versus primary bladder tumors. **(C)** The PCA analysis showing that most recurrent tumors were separated from the primary tumors. **(D)** Gene set enrichment analysis (GSEA) showing that gene expression levels of “EMT” and “Inflammatory Response” were positively correlation with the recurrent tumors, while genes of “Myc target v2” and “P53 pathways” were negatively correlated with the recurrent tumors.

We performed differential expression analysis (DEA) with the R packages of DESeq2 bioconductor to identify the differentially expressed genes (DEGs) between the primary BC group and recurrent BC. The selection criteria were | Log2Foldchange | > 1 and P-FDR < 0.05. The volcano plot was created using the R ggplot2 package (Figure 1A). The top 30 significantly differentially expressed genes in recurrent BC and primary BC were identified and plotted on a heatmap (Figure 1B); red indicates high-expression genes in the heatmap. Recurrent tumors were efficiently differentiated from primary tumors using Principal Component Analysis (PCA) (Figure 1C). Gene set enrichment analysis (GSEA) was performed to confirm the differences in pathways between recurrent BC and primary BC. The results revealed a positive correlation between gene expression levels of “Epithelial-Mesenchymal Transition (EMT)” and “Inflammatory Response” and recurrent BC. In contrast, genes associated with the “Myc target v2” and “P53 pathways” were negatively correlated with recurrent BC (Figure 1D).

### 3.3 Gene ontology functional enrichment analyses for DEGs

**FIGURE 2:**
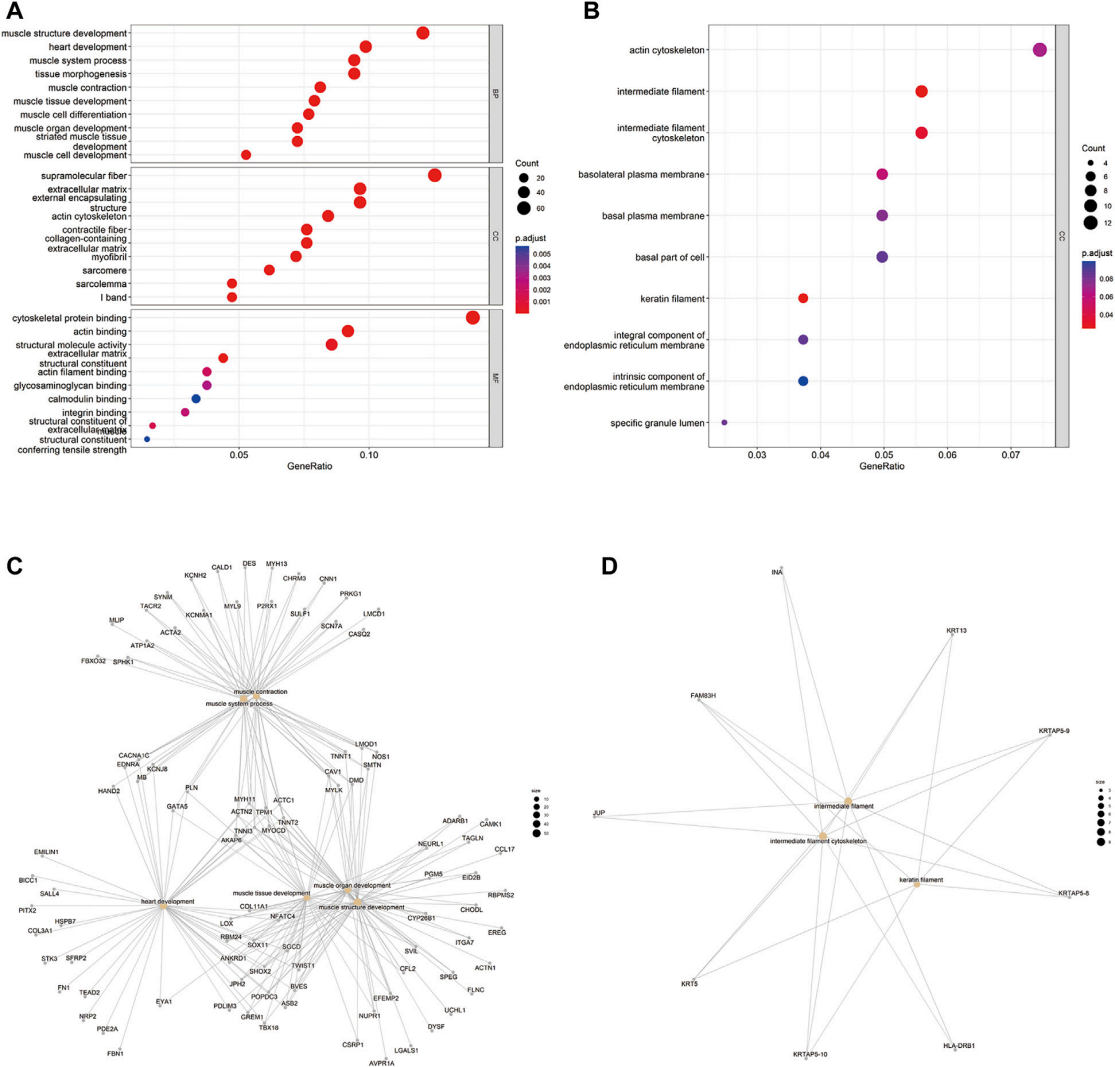
GO term enrichment analysis results of DEGs of 5 recurrent vs. 5 primary tumors. **(A)** GO term function prediction showing the gene ontology enrichment of DEGs up-regulated in recurrent tumors. The ordinate represents GO term description information, BP: biological process, CC: cell composition, MF: molecular function, the abscissa is the number of differential genes enriched to the term, *p* < 0.05. The color of the *p*-value ranges from blue (0.005) to red (0.001). **(B)** GO term function prediction showing the gene ontology enrichment of DEGs downregulated in recurrent tumors. The ordinate represents GO term description information, CC: cell composition, the abscissa is the number of differential genes enriched to the term, *p* < 0.05. The color of the *p*-value ranges from blue (0.06) to red (0.04). **(C**,**D)** Cneplot showing the genes involved in the top 9 GO terms enrichment results of the DEGs (left, up-regulated in recurrent tumors; right, downregulated in recurrent tumors).

EnrichR and clusterprofiler were used to conduct GO functional enrichment analyses to determine gene function in the primary bladder cancer group versus the recurrent bladder cancer group. The gene ontology includes biological process (BP), cellular components (CC), and molecular functions (MF). The top 10 most significant GO terms from each group were displayed. The GO enrichment analysis showed that genes up-regulated in recurrent BC were predominantly involved in BC, CC, and MF (Figure 2A). However, genes down-regulated in recurrent BC were mostly found in CC (Figure 2B). Up-regulated DEGs were enriched mainly in muscle structure development of BPs, the supramolecular fiber of CCs, and cytoskeletal protein binding of MFs, while down-regulated DEGs were enriched mainly in intermediate filament and intermediate filament cytoskeleton of CCs. Subsequently, we used a cnplot to show the GO terms enrichment results for the DEGs (Figures 2C,D). Among them, MYH11, ACTC1, ACTN2, TPM1, TNNT2, TNNI3, MYOCD, and AKAP6, which were up-regulated in recurrent BC, were enriched in 6 GO terms of BPs. FAM83H, KRTAP5-8, KRTAP5-9, and KRTAP5-10, down-regulated in recurrent BC, were enriched in 3 GO terms of CCs.

### 3.4 Differential expression analysis of miRNA-seq

**FIGURE 3:**
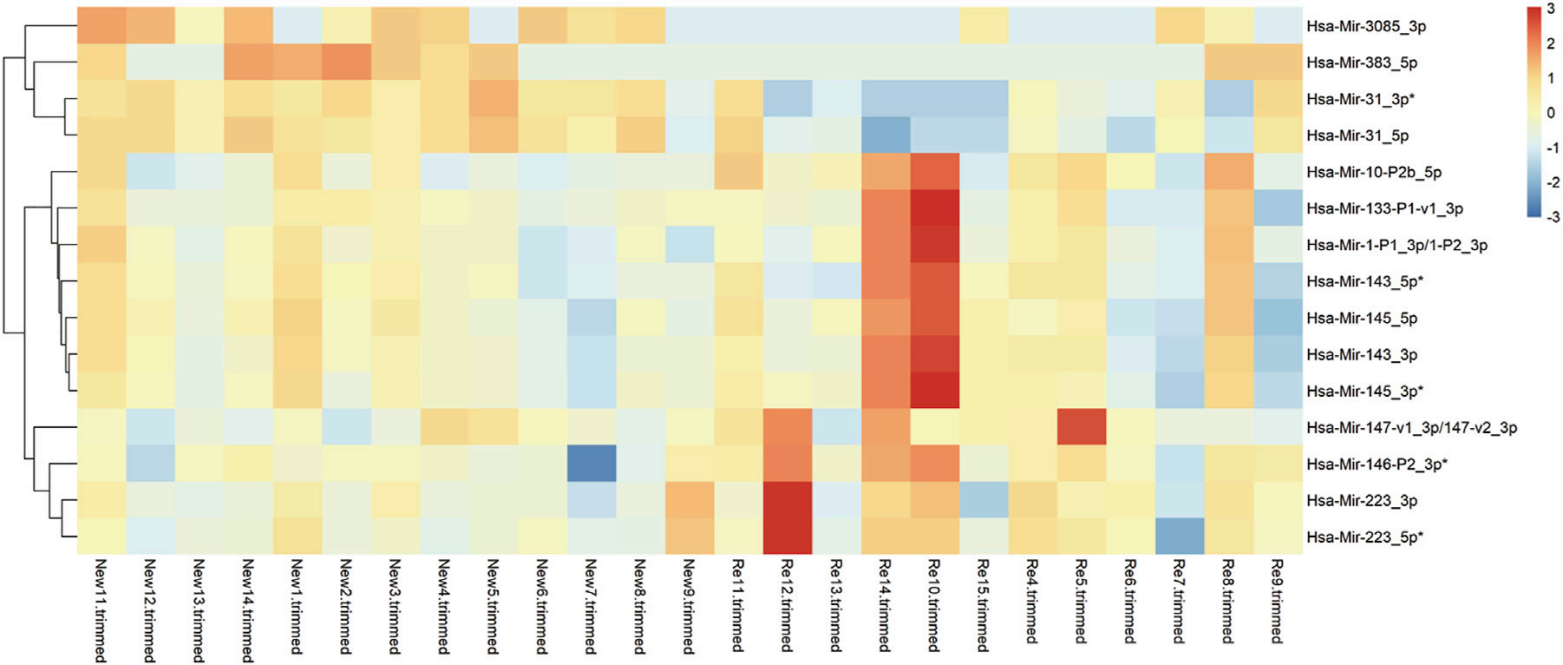
Overview of miRNA-seq results of 12 recurrent tumors vs. 13 primary tumors. **(A)** A Heatmap showing the top 15 differentially expressed miRNAs in 12 recurrent vs. 13 primary tumors. In recurrent BC, miR-3085-3p and miR-31-5p were significantly down-regulated, while miR-223-3p and miR-146-3p were up-regulated.

MiRNA is an extensively existing non-coding single-stranded RNA, which regulates gene expression at the post-transcriptional level by inhibiting the translation of messenger RNA (mRNA) or promoting mRNA degradation. To analyze the differential changes in miRNA expression levels in primary and recurrent BC, We performed differential expression analysis on the miRNA-seq sequencing results of 12 cases of recurrent bladder cancer and 13 cases of primary bladder cancer and plotted the top 15 differentially expressed miRNAs on a heat map (Figure 3). The cluster analysis revealed significant differences in miRNA expression between recurrent and primary BC. In recurrent BC, miR-3085-3p and miR-31-5p were significantly down-regulated, while miR-223-3p and miR-146-3p were up-regulated.

### 3.5 Identification and analysis of miRNAs’ A-to-I editing events between primary and recurrent bladder cancer

**FIGURE 4:**
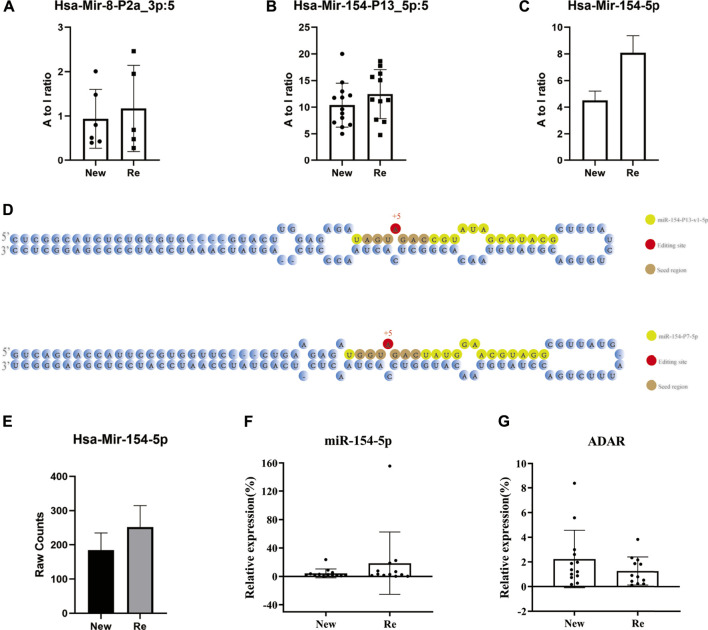
Overview of miRNA editing events of 12 recurrent tumors vs. 13 primary tumors. **(A**–**C)** Bar plots showing miRNA editing A-to-I ratio levels of three different miRNAs in both primary and recurrent tumors, with hyper edited in recurrent BC (*p*-value<0.05). Hsa-Mir-154-5p exhibited the most significant difference.*: *p*-value < 0.05. **(D)** Identification of edited mature miR-154. The 5′ end of the mature miRNA is counted as +1, and the editing site is indicated by the number highlighted in red. The seed region is characterised by highlighted in brown. **(E)** A Bar plot showing the raw count level of mir-154-5p in both primary and recurrent tumors. **(F**,**G)** RT-qPCR result for miR-154-5p and ADAR. In recurrent tumors, miR-154-5p expression levels were slightly upregulated (*p*-value = 0.2567), while ADAR expression levels were downregulated (*p*-value = 0.2072).

We identified and compared miRNAs A-to-I editing events in recurrent and primary BC, and the A-to-I editing ratio in three types of miRNAs was displayed using bar plots (Figures 4A–C). The bar plots showed that Hsa-Mir-8-P2a_3p, Hsa-Mir-154-P13_5p, and Hsa-Mir-154-5p had A-TO-I editing in primary and recurrent BC, with hyper edited in recurrent BC (*p*-value<0.05). Hsa-Mir-154-5p exhibited the most significant difference. Subsequently, we identified the editing sites for miR-154-5p. Both miR-154-P13-5p and miR-154-P7-5p underwent A-to-I editing at the fifth base, and the editing site was located within the seed region (Figure 4D).

To rule out the effect of miRNA expression level, we investigated the raw count level of miR-154-5p in both primary and recurrent tumors and validated it by RT-qPCR. The results showed that the miR-154-5p expression level was slightly up-regulated in recurrent BC (*p*-value = 0.2567) (Figures 4E,F). This part of the analysis indicated that differential levels of miRNA editing might not be a function of differential expression.

In previous studies, ADAR enzymes were found to mediate A-to-I RNA editing (Eisenberg and Levanon, 2018; Yao et al., 2019). ADAR-mediated A-to-I RNA editing can affect miRNA-controlled pathways by interfering with miRNA maturation steps as well as with miRNA targeting (Nishikura, 2016). To further investigate the causes of miRNA editing events in bladder cancer, we compared the expression levels of the ADAR (ADAR1) gene in recurrent and primary BC using RT-qPCR. Surprisingly, the expression level of ADAR did not change significantly in recurrent BC (Slightly down-regulated in recurrent BC, *p*-value = 0.2072) (Figure 4G). One possible reason is that we only examined the expression of the ADAR gene and did not verify the changes in ADAR enzyme expression in BC. Second, the regulation of A-TO-I editing by ADAR is a complex process that may be influenced by the physiological environment as well as competitive inhibition of ADAR2 (Buchumenski et al., 2017; Vesely and Jantsch, 2021).

### 3.6 A-to-I editing of miR-154-5p may regulate the expression level of the subsequent miRNA targets

**FIGURE 5:**
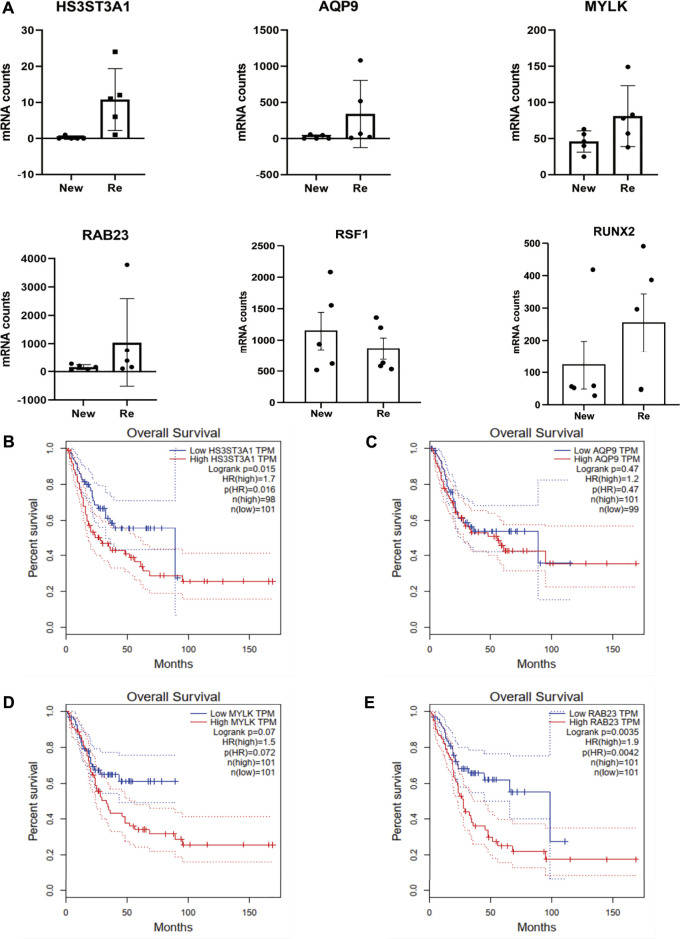
Expression levels of mir-154-5p targets are potentially regulated by the editing of mir-154-5p other than the expression level of mir-154-5p. **(A)** A Bar plot showing the mRNA expression level of mir-154 targets. HS3ST3A1, AQP9, MYLK, and RAB23 are significantly differentially expressed in recurrent vs. primary tumors, with a *p*-value after FDR correction <0.001. **(B**–**E)** Survival plots of the four top targets (high expression; > 50% vs. low expression <50%). Survival data were extracted from TCGA bladder cancer cohorts.

Since the role of miRNA in translational repression is conferred by sequence complementarity between the miRNA seed region and the complementary seed region in the target mRNA, editing in the seed region can determine changes in miRNA target repertoires by even a single nucleotide substitution (Choudhury et al., 2012; Cesarini et al., 2018). We used miRDB and TargetScanHuman to identify the top four targets of miR-154-5p, HS3ST3A1, AQP9, MYLK, and RAB23 and compared their expression level in recurrent BC and primary BC. The results showed that all four targets were differentially expressed in recurrent and primary BC, with recurrent BC having significantly higher expression levels (*p*-value after FDR correction <0.001) (Figure 5A). Subsequently, we analyzed other reported miR-154-5p target genes, including RSF1 and RUNX2 (Zhao et al., 2017). Our results showed that the expression levels of miR-154-5p and RSF1 were up-regulated in recurrent bladder cancer while RUNX2 was down-regulated, and the differences were insignificant (Figure 5A). Because there was no significant difference in the expression level of miR-154-5p (Figures 4E,F), we hypothesized that the expression level of the miR-154-5p targets might be regulated by editing of mir-154-5p in addition to the expression level of miR-154-5p. Next, we downloaded samples expressing low and high levels of HS3ST3A1 [n (low) = 101, n (high) = 98], AQP9 [n (low) = 99, n (high) = 101], MYLK [n (low) = 101, n (high) = 101], and RAB23 [n (low) = 101, n (high) = 101] in bladder cancer cohorts from the TCGA database for survival analysis (high expression; > 50% vs. low expression <50%). The survival analysis showed that BC patients expressing high levels of HS3ST3A1 and RAB23 had a poor prognosis (HS3ST3A1 *p* < 0.005, RAB23 *p* < 0.005, by log-rank test) (Figures 5B–E).

### 3.7 MiRNA editing events in BC are associated with consensus molecular subtype classification

**FIGURE 6:**
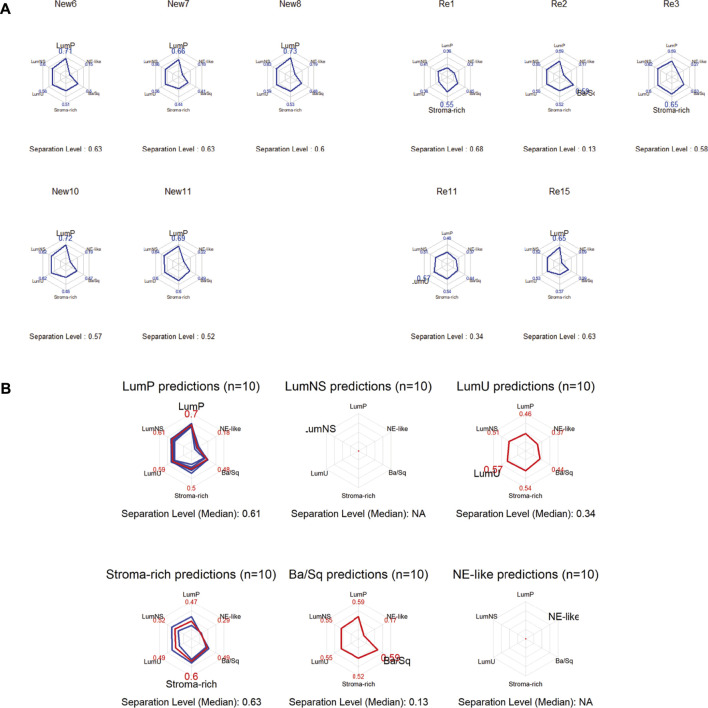
miRNA editing events are associated with CMS classification in bladder tumors. **(A)** Individual CMS classification results of 5 recurrent and 5 primary tumors. **(B)** Overview of CMS prediction of all 10 samples.

Bladder cancers are classified into six CMS classes: luminal papillary (LumP), luminal nonspecified (LumNS), luminal unstable (LumU), stroma-rich, basal/squamous (Ba/Sq), and neuroendocrine-like (NE-like) (Kamoun et al., 2020). Using the single-sample mRNA classifier from the Consensus MIBC package based on R (4.1.0), we identified the CMS classification of five recurrent and five primary BC; LumP had the most samples (60%). The remaining 40% were stroma-rich (20%), Ba/Sq (10%), and LumU (10%) (Figure 6A). The results of CMS prediction of all ten samples were shown in a radar plot (Figure 6B). We then examined miR-154 editing events in 10 samples (Table 2). Five of the 6 CMS LumP samples showed miR-154 editing events. However, miR-154 editing events were not found in other CMS types (Table 3). Therefore, we hypothesize that miR-154 editing events are associated with CMS typing of bladder cancer.

**TABLE 2:** Overview of all miRNA editing events of 10 cases with mRNA-seq results.

miRNA: Position	Sample	A-to-Ipercentage
Hsa-Mir-154-P13_5p:5	Re15	4.78
Hsa-Mir-154-P9_3p:4	Re15	1.16
Hsa-Mir-589_3p*:6	Re11	63.64
Hsa-Mir-154-P13_5p:5	Re11	7.67
Hsa-Mir-34-P3c_5p:16	Re11	3.77
Hsa-Mir-154-P9_3p:4	Re11	2.9
Hsa-Mir-154-P13_5p:5	New8	6.25
Hsa-Mir-27-P1_5p*:1	New8	5.88
Hsa-Mir-154-P9_3p:4	New8	1.75
Hsa-Mir-8-P2a_3p:5	New8	0.51
Hsa-Mir-154-P13_5p:5	New7	11.76
Hsa-Mir-154-P9_3p:4	New7	5
Hsa-Mir-8-P2a_3p:5	New7	0.4
Hsa-Mir-154-P13_5p:5	New6	20
Hsa-Mir-1301_3p:5	New6	6.45
Hsa-Mir-8-P2a_3p:5	New6	0.8
Hsa-Mir-154-P13_5p:5	New11	12.22
Hsa-Mir-154-P9_3p:4	New11	2.33
Hsa-Mir-10-P2b_5p:1	New11	0.72

**TABLE 3:** miR-154 editing events in 10 cases that have mRNA-seq results; 5 of the 6 LumP cases have miR-154 editing events.

Total Case	LumP	Mir-154-editing
10	6	5
	Others	miR-154 editing
	4	0

## 4 Discussion

With the advancement of sequencing technology, more and more miRNA editing events have been identified in various cancers. In 2014, Jing Gong et al. confirmed 14 A-to-I editing loci through systematic analysis of 410 human smRNA-seq datasets, 11 of which were significantly different in the liver, breast, and lung cancers, and 4 of the 11 loci were up-regulated, while 7 were down-regulated in tumor tissues (Gong et al., 2014). Yishay Pinto extensively documented miRNA editing in 32 cancer types and 10,593 human samples of normal controls in 2018 and presented a list of 58 miRNA editors with high confidence, and 24% of loci exhibited significant differences in tumor tissues compared to normal tissues (Pinto et al., 2018). Similarly, Yumeng Wang et al. analyzed 8595 TCGA samples from 20 major cancer types and found widespread A (Adenosine)-to-I (Creatinine) editing in bladder uroepithelial carcinoma, and edited miR-381 was associated with tumor stage. In contrast, miR-379 and miR-411 were associated with tumor subtype, but none was linked to bladder cancer recurrence and prognosis (Wang et al., 2017). Because bladder cancer is characterized by heterochrony, heterotopy, high recurrence, multiple tumors, progression to invasive bladder cancer, and metastasis, miRNA editing research can help further elucidate the molecular mechanisms of bladder cancer and serve as a biomarker to predict bladder cancer progression, recurrence, and patient prognosis.

In this study, we performed RNA sequencing on tissue specimens from 25 BC patients (12 recurrent BC and 13 primary BC) and performed a comprehensive analysis of mRNAseq and miRNAseq to determine miRNA editing in bladder cancer. First, differential changes in mRNA expression levels between primary BC and recurrent BC were discovered using mRNAseq differential expression and enrichment analysis, which supported tumor progression *via* associated signalling pathways. Second, we screened three highly edited miRNA loci in recurrent BC and found that miRNA editing might influence the expression levels of downstream targets. Survival analysis revealed that changes in downstream target expression levels were associated with a worse patient prognosis. Finally, we discovered that miRNA editing events appeared to correspond with CMS classification of bladder cancer.

The mRNAseq sequence analysis revealed significant differences between recurrent and primary BC, which may contribute to cancer progression and recurrence through changes in associated pathways. We screened the top 30 differential genes from mRNAseq of 10 cases (5 primary LG NMIBC and 5 recurrent HG MIBC), and the gene set enrichment analysis (GSEA) results revealed that Epithelial-Mesenchymal Transition (EMT)" and “Inflammatory Response” were up-regulated in recurrent BC, while “Myc target v2″ and “P53 pathways " were down-regulated in recurrent BC. EMT is a complicated process that involves transitions from an epithelial to a mesenchymal phenotype, resulting in functional changes in cell migration and invasion. Similarly, in previous work, EMT was found to be up-regulated in multiple carcinogeneses, contributing to cancer progression and metastasis (Yang et al., 2020). In BC, EMT promotes muscle infiltration, recurrence, and metastasis via a variety of mechanisms (Wang et al., 2021; Na et al., 2022). The inflammatory response has been proven to be important in various stages of tumor formation, including tumorigenesis, growth, progression, and metastasis (Greten and Grivennikov, 2019). By investigating the kinetics of inflammatory response during BBN-induced bladder carcinogenesis in mice, Marina Degoricija et al. discovered that up-regulated IFNγ genes and immunosuppressive genes mediated tumor immune escape, leading to bladder cancer invasion and progression (Degoricija et al., 2019). P53 pathways are tumor suppressor pathways, and gene mutations result in impaired P53 function (Whibley et al., 2009; Hernández Borrero and El-Deiry, 2021). Soonbum Park et al. found that knocking down the P53 tumor suppressor gene resulted in a shift from non-muscle-invasive to muscle-invasive BC, as well as an increase in cancer-related molecular pathways, including pro-inflammatory pathways (Park et al., 2021). Moreover, increased Myc target v2 expression promotes tumor cell progression and invasion (Schulze et al., 2020). However, Myc target v2 was shown to be down-regulated in recurrent MIBC in our investigation, which is consistent with the findings of Fragkoulis et al. in NMIBC (LG) and MIBC (HG) (Fragkoulis et al., 2021). Further GO functional enrichment analyses revealed that up-regulated DEGs were primarily associated with muscle structure development (BP), supramolecular fiber (CC), and cytoskeletal protein binding (MF), whereas down-regulated DEGs were associated with intermediate filament and intermediate filament cytoskeleton of CCs. Based on mRNA sequence analysis, we discovered differentially altered mRNAs in recurrent bladder cancer that promoted cancer invasion and progression via related pathways, providing support for previous findings in BC.

MiRNAs are differentially expressed in primary and recurrent BC. Based on miRNA sequencing, we found that in recurrent BC, miR-3085-3p and miR-31-5p were significantly down-regulated while miR-223-3p and miR-146-3p were up-regulated. MiR-31-5p has been shown to exert tumor suppressive effects in bladder cancer by modulating downstream targets, including RAB27A and KRT6A. RAB27A and KRT6A promote proliferation and adhesion of bladder tumors and negatively correlate with miR-31-5p expression levels (Bi et al., 2018; Chen et al., 2022). In our results, miR-31-5p was down-regulated in recurrent BC, possibly impairing its tumor suppressive effect. MiR-3085-3p can promote catabolism by decreasing stromal gene expression, enhancing IL-1 signaling and reducing TGFβ signaling< which has been shown to play a role in cartilage development, homeostasis and breakdown during osteoarthritis (Le et al., 2020). However, miR-3085-3p has not yet been reported in BC. In recurrent BC, we observed a significant up-regulation of miR-146-3p and miR-223-3p. In contrast to our results, by comparing bladder carcinoma tissue and adjacent non-tumor tissue, Guo et al. found that miR-233-3p expression was down-regulated in BC tissues and that low expression was associated with poor patient prognosis and noted that miR-223-3p might bind to the 3′-UTR of nuclear receptor coactivator 1 messenger RNA to inhibit its protein translation in bladder carcinoma cells (Guo et al., 2017). Interestingly, Xue et al. performed a transcriptome analysis of low-grade, high-grade uroepithelial cancer tissue and found that miR-223-3p expressed highly in high-grade BC and positively correlated with M2 macrophage infiltration, which is similar to our study results (Xue et al., 2019). Similarly, by analyzing the differential changes in bladder cancer miRNAs, Baumgart et al. found that miR-146b-5p and miR-223-3p were up-regulated in BC, with miR-146b-5p being significantly associated with high-grade tumors (Baumgart et al., 2019). In conclusion, we identified differentially expressed miRNAs in recurrent BC. However, the regulation of tumors by miRNAs is a complex series of processes, and further studies are needed to determine their specific roles.

A-to-I miRNA editing was significantly altered in recurrent BC, which may regulate the expression levels of downstream targets and correlate with patient prognosis. We discovered three highly edited miRNAs in recurrent BC, namely Hsa-Mir-8-P2a_3p, Hsa-Mir-154-P13_5p, and Hsa-Mir-154-5p, with Hsa-Mir-154-5p showing the most significant difference (*p* < 0.05). Subsequently, we identified the editing sites for miR-154-5p and found that the editing sites were located within the seed region. Mounting evidence shows that miR-154-5p promotes cell apoptosis and inhibits tumor growth, invasion, and metastasis in cancers such as Nasopharyngeal Carcinoma, Retino Blastoma, and Osteo Sarcoma (Chen et al., 2020; Liu et al., 2020; Tian et al., 2020). Since the levels of miR-154-5P expression did not differ significantly between primary and recurrent BC, we propose that the high editing of miR-154-5p may result in differential expression of downstream targets. We evaluated the expression levels of the top four targets downstream of miR-154-5p (HS3ST3A1, AQP9, MYLK, and RAB23) and found that their expression was significantly higher in recurrent BC (*p*-value after FDR correction <0.001). Then we analyzed the expression levels of previously reported miR-154 targets, including RSF1 and RUNX2. Our results showed that RSF1 was up-regulated in recurrent BC while RUNX2 was down-regulated. The differences were not significant. In contrast to our study, Zhao et al. showed that miR-154 expression was significantly down-regulated in bladder cancer and negatively correlated with RSF1 and RUNX2 expression levels (Zhao et al., 2017). This may be due to the editing of the miR-154-5p seed region resulting in differential expression of its downstream targets. Just as Warnefors et al. found that miRNA editing causes dramatic changes in target specificity or expression levels in mammalian studies, which is consistent with our findings (Warnefors et al., 2014). Previous research has linked aberrant expression of HS3ST3A1, AQP9, MYLK, and RAB23 to cancer formation, invasion, and prognosis. The gene HS3ST3A1 encodes Heparan sulfate glucosamine 3-O-sulfotransferase 3A1, which is involved in a late modification step during acetyl Heparan sulfate (HS) biosynthesis. HS3ST3A1 expression is higher in lung cancer compared to normal tissue (Nakano et al., 2012), however, in HER2+ patients with breast cancer, high levels of HS3ST3A expression are associated with reduced recurrence-free survival (Mao et al., 2016); AQP9 encodes the aquaporin 9, which is involved in processes related to cell migration, angiogenesis, and tumor growth (Nico and Ribatti, 2011). Furthermore, in renal clear cell carcinoma (KIRC), shorter survival was found in the AQP9 high expression group than in the low expression group (Jing et al., 2021). Elsewhere, Ning X et al. discovered MYLK overexpression in bladder cancer and linked it to prognosis (Ning and Deng, 2017). In a study by Jiang Y et al., RAB23 was shown to be over-expressed in bladder cancer, and there was a significant correlation between RAB23 and depth of tumor invasion (*p* = 0.0027) (Jiang et al., 2016). Our survival analysis revealed that BC patients expressing high levels of HS3ST3A1 and RAB23 had a poor prognosis (HS3ST3A1 *p* < 0.005, RAB23 *p* < 0.005, by log-rank test). Of note, we first identified highly edited miRNA loci in recurrent BC using miRNA sequencing analysis, A-to-I editing analysis, downstream analysis and survival analysis and found that the expression levels of miRNA downstream targets were potentially largely regulated by miRNA editing and correlated with prognosis.

In addition, we discovered that miRNA editing appears to be linked to the CMS classification of bladder cancer. The six CMS classes of BC include LumP, LumNS, LumU, stroma-rich, Ba/Sq, and NE-like (Kamoun et al., 2020). By extracting and organizing the expression matrices of each bladder cancer sample, we found that 5 of the 6 samples of CMS LumP samples had miR-154 editing events. However, miR-154 editing events were not detected in other CMS types. Therefore, we hypothesized a link between miR-154 editing events and CMS classification of bladder cancer. Of note, in a study by Aurélie Kamoun et al., the FGFR3 signature was robustly and specifically in LumP tumors (*p* < 0.001), implying that FGFR3-targeted therapies warrant investigation in patients with this consensus class of tumors (Kamoun et al., 2020). More and more research is indicating that molecular subtypes may play an important part in the decision-making process of individualized BC treatment (Robertson et al., 2020; Chu et al., 2021). We believe that the finding of this study have some reference value and can help us understand the potential mechanism of bladder cancer classification and guide individualized treatment.

However, there are some limitations to our study. Because A-to-I miRNA editing is a series of complex processes, and although the significantly edited miRNA has been proven in recurrent bladder cancer, we have not deeply explored the causes of this phenomenon. Furthermore, although we first identified miRNA editing events in recurrent bladder cancer and hypothesized that miRNA editing would potentially affect the expression levels of its downstream targets. However, miRNA editing and deregulated expression of the target genes could be two independent events. We need to perform follow-up experiments to verify the specific effects of miRNA editing on its downstream targets. Finally, a larger sample volume is required to confirm the association between miRNA editing and bladder cancer CMS classification.

## 5 Conclusion

This work discovered the differentially edited miRNA in primary and recurrent bladder cancer. miR-154-5p was significantly edited in the recurrent bladder cancer and linked with patient prognosis, indicating that A-to-I editing of miR-154-5p could be a promising target for bladder cancer treatment. This can help in overcoming the limits of current therapeutic approaches. Furthermore, miR-154 editing events are potentially significant to bladder cancer CMS classification. These findings serve as a foundation for further research into complex molecular pathways in recurrent bladder cancer.

## Data Availability

RNA-seq data has been deposited to GEO (https://www.ncbi.nlm.nih.gov/geo/query/acc.cgi?acc=GSE212139; Accession number: GSE212139.
